# Pharmacological mechanism underlying anti-inflammatory properties of two structurally divergent coumarins through the inhibition of pro-inflammatory enzymes and cytokines

**DOI:** 10.1186/s12950-015-0087-y

**Published:** 2015-07-29

**Authors:** Salman Khan, Omer Shehzad, Mao-Sheng Cheng, Rui-Juan Li, Yeong Shik Kim

**Affiliations:** The Alan Edwards Centre for Research on Pain, McGill University, Montreal, QC H3A 0G1 Canada; College of Pharmacy, Seoul National University, Gwanak-gu, Seoul 151-742 South Korea; Department of Pharmacy, Abdul Wali Khan University, Mardan, Pakistan; School of Pharmaceutical Engineering, Shenyang Pharmaceutical University, Shenyang, 110016 China

**Keywords:** Calipteryxin, (3*’S*,4*’S*)-3’,4’-disenecioyloxy-3’,4’-dihydroseselin, NF-κB, MAPK, Akt, *Seseli recinosum*

## Abstract

**Background:**

The aim of the present study is to investigate the effects of two structurally divergent coumarins, calipteryxin (1) and (3*’S*,4*’S*)-3’,4’-disenecioyloxy-3’,4’-dihydroseselin (2) from *Seseli recinosum*, in lipopolysaccharide (LPS)-stimulated murine macrophages.

**Methods:**

The nitrite production was evaluated using Griess reagent. The protein and mRNA expression levels were investigated through Western blot and quantitative real time-PCR analyses. The NF-κB and AP-1 DNA-binding activities were assessed using an electrophoretic mobility shift assay. The docking studies were performed with Glide XP in Schrödinger suite (version 2013).

**Results:**

The results of the present study revealed that calipteryxin (1) and (3*’S*,4*’S*)-3’,4’-disenecioyloxy-3’,4’-dihydroseselin (2) treatment showed potent inhibitory effects on pro-inflammatory enzymes and cytokines associated with molecular signaling pathways. Treatment with calipteryxin and (3*’S*,4*’S*)-3’,4’-disenecioyloxy-3’,4’-dihydroseselin also decreased the production of nitric oxide (NO), tumor necrosis factor alpha (TNF-α) and interleukin-1 beta (IL-1β) in a dose-dependent manner. Additionally, both coumarins inhibited the LPS-induced protein and mRNA expression levels of nitric oxide synthase (iNOS) and cyclooxygenase-2 (COX-2) in RAW264.7 cells. To explore the potential mechanisms underlying the inhibitory activity of coumarin derivatives, the protein signaling pathways for NF-κB, mitogen-activated protein kinase (MAPK) and Akt were examined. Calipteryxin and (3*’S*,4*’S*)-3’,4’-disenecioyloxy-3’,4’-dihydroseselin markedly reduced the LPS-stimulated phosphorylation of IKKα/β, p-IκBα and IκBα degradation as well as the nuclear translocation of the p65 subunit of pro-inflammatory transcription factor NF-κB. In addition, calipteryxin and (3*’S*,4*’S*)-3’,4’-disenecioyloxy-3’,4’-dihydroseselin) considerably inhibited the LPS-induced expression of ERK, c-Jun *N*-terminal kinase (JNK), p38 and Akt proteins. Furthermore, both coumarins significantly inhibited c-Jun expression in the nucleus.

**Conclusions:**

Taken together, these results support the therapeutic potential and molecular mechanism of calipteryxin and (3*’S*,4*’S*)-3’,4’-disenecioyloxy-3’,4’-dihydroseselin associated with inflammatory diseases.

**Electronic supplementary material:**

The online version of this article (doi:10.1186/s12950-015-0087-y) contains supplementary material, which is available to authorized users.

## Background

Considerable amounts of pro-inflammatory mediators and pro-inflammatory cytokines are released at injury sites during inflammation [1]. These pro-inflammatory mediators respond to numerous stimuli, including bacterial lipopolysaccharide (LPS), cytokines, and UV irradiation, which modulate to their effects by inducing the activation of NF-κB and AP-1 [2].

NF-κB activates a number of molecules involved in the inflammatory response, including iNOS, COX-2, TNF-α, IL-1β, and IL-6 [1]. The production of these mediators and cytokines through NF-κB stimulation might reflect the extent of inflammation and has been suggested as a measure to evaluate the effects of anti-inflammatory agents on the inflammatory process. NF-κB signaling is activated through two diverse pathways: the canonical (classical) pathway and the non-canonical pathway [3]. NF-κB signaling through canonical or non-canonical pathways involves the expression of multiple genes [4, 5]. The canonical pathway involves the IκBα kinase (IKK) complex, while the non-canonical pathway involves NF-κB inducing kinase (NIK), which recruits IKK to p100 and subsequently activates IKK [4]. In response to inflammation, the p50 and p65 subunits of NF-κB are translocated through the canonical pathway, whereas the p52-containing RelB heterodimer of NF-κB is released and translocated through the non-canonical pathway [4, 5].

Mitogen-activated protein (MAP) kinases and PI3k/Akt signaling pathways play essential roles in inflammation and tissue remodeling [6, 7]. Consequently, the inhibition of MAP kinases produces anti-inflammatory effects against various inflammatory diseases [6]. However, several studies have demonstrated that the activation of MAP kinases suppresses inflammatory reactions, such as bacterial LPS-induced cytokine production in macrophages.

In the present study, two structurally divergent coumarin derivatives, calipteryxin (1) and (3*’S*,4*’S*)-3’,4’-disenecioyloxy-3’,4’-dihydroseselin (2) isolated from *Seseli resinosum*, were studied in terms of LPS-induced inflammatory signaling. The genus *Seseli* L. belongs to the Apiaceae family, which comprises aromatic herbs used as foods, spices, condiments and ornamentals [8]. This herb has various pharmacological applications, such as anthelmintic, carminative, stomachic and stimulant properties [8]. It has been reported that *Seseli* plants show significant and dose-dependent anti-inflammatory activity and analgesic effects in carrageenan-induced acute inflammation in rats [8]. However, only a few pharmacological and biological activity studies concerning the single component from *Seseli* have been reported. Therefore, the aim of the present study was to examine LPS-induced inflammatory signaling in murin macrophages. Moreover, a comparative investigation was performed between two coumarin type compounds.

## Methods

### Isolation and purification of calipteryxin and (3’*S*,4’*S*)-3’,4’-disenecioyloxy-3’, 4’-dihydroseselin

**Fig. 1:**
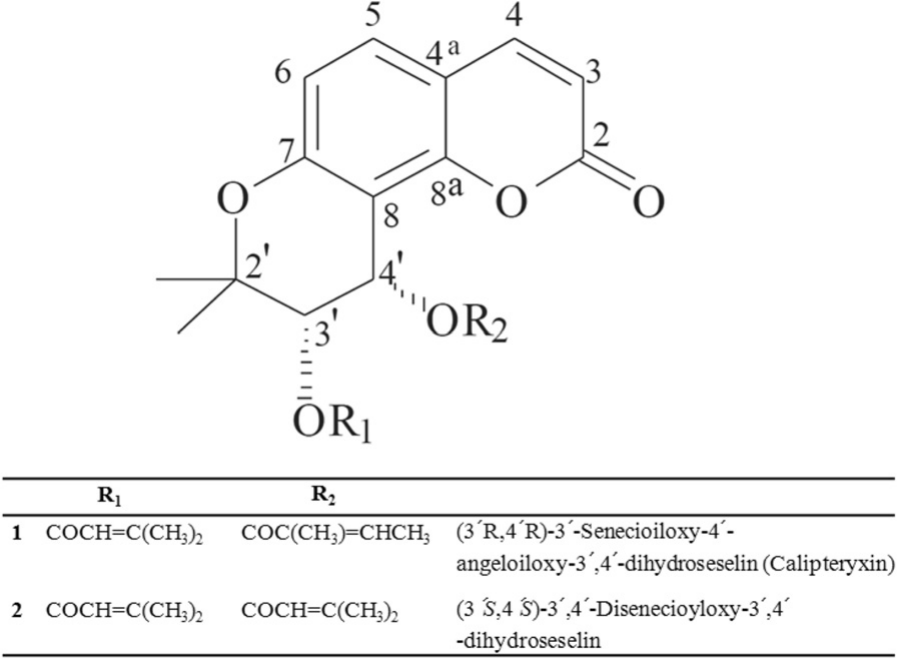
Chemical structures of calipteryxin and (3*’S*,4*’S*)-3’,4’-disenecioyloxy-3’,4’-dihydroseselin

The separation and purification of calipteryxin and (3*’S,*4’*S)-*3’ ,4’-disenecioyloxy-3’ ,4’-dihydroseselin, from *Seseli resinosum* were performed using counter-current chromatography coupled with an evaporative light scattering detector (CCC-ELSD). Based on optimum *K*_D_ values of the target compounds, a two-phase solvent gradient system comprising n-hexane/ethyl acetate/methanol/water at a volume ratio of 3:2:3:2 and 2:1:2:1 was used. The peak fractions were collected using CCC-ELSD chromatography. The collected peak fractions were dried under reduced pressure and analyzed through HPLC-ELSD. The results confirmed that the purity of each compound was ≥ 99 %. Moreover, the structures of calipteryxin and (3*’S,*4’*S)-*3’ ,4’-disenecioyloxy-3’,4’-dihydroseselin (Fig. 1) were confirmed based on ESI-MS/MS, ^1^H-NMR and ^13^C-NMR data, as described in previous studies [9].

### Cells and culture medium

RAW 264.7 murine macrophages were obtained from the American Type Culture Collection (Manassas, VA). These macrophages were maintained and subcultured according to a previously described procedure [5].

### Cell Viability and nitric oxide determination

The cell viability was determined using an MTT assay according to Khan et al. [5]. The RAW 264.7 cells were treated with various concentrations (2.5 ~ 30 μM) of calipteryxin and (3*’S*,4*’S*)-3’ ,4’-disenecioyloxy-3’ ,4’-dihydroseselin or vehicle alone for 2 h prior to LPS (1 μg/ml) stimulation and subsequently incubated at 37 °C for an additional 18 h. The NO was measured using the Griess reaction as previously described [5].

### Western immunoblot analysis

The Western blot procedure was performed according to Khan et al., with some modifications [5].

### RNA extraction and quantitative real-time (RT) PCR

RT-PCR was performed with total RNA extracted using easyBlue™ according to the manufacturer’s instructions (Sigma-Aldrich, St. Louis, MO). The purity and concentrations of RNA were determined using an ND-1000 spectrophotometer (Nanodrop Technologies, Wilmington, DE). All RNA samples were stored at −80 °C until further analysis. Total RNA (1 μg) was converted to cDNA through RT-PCR (Genius FGEN05TD, Teche, England) using the iScript™ cDNA Synthesis Kit (BIO-RAD, Hercules, CA) under the following conditions: 25 °C for 5 min, 42 °C for 30 min and 85 °C for 5 min. Quantitative real-time polymerase chain reaction (qRT-PCR) analysis was performed using an Applied Biosystems 7300 Real-Time PCR system and software (Applied Biosystem, Carlsbad, CA). qRT-PCR was conducted in 0.2 ml PCR tubes with forward and reverse primers and the SYBR green working solution (iTaq™ Universal SYBR Green Supermix, BIO-RAD, Hercules, CA), using customer PCR master mix under the following conditions: 95 °C for 30 min, followed by 40 cycles of 95 °C for 15 s, 55 °C for 20 s and 72 °C for 35 s. The melting point, optimal conditions and specificity of the reactions were determined. The sequences of the PCR primers were previously described [10, 11]. The sense and antisense primers for iNOS were 5’CCCTTCCGAAGTTTCTGGCAGC-3’ and 5’-GGCTGTCAGAGCCTCGTGGCTT-3’, respectively. The sense and antisense primers for COX-2 were 5’-GGAGAGACTATCAAGATAGTGATC- 3’ and 5’-ATGGTCAGTAGACTTTTACA-GCTC-3’ , respectively. The following primers were used for; TNF-α, sense primer, 5’-AGC ACA GAA AGC ATG ATC CG-3’ and antisense primer, 5’-CTG ATG AGA GGG AGG CCA TT-3’; and for IL-1β, sense primer, 5’-ACCT GCT GGT GTG TGA CGT T-3’, and antisense primer, 5’-TCG TTG CTT GGT TCT CCT TG-3’. The sense and antisense primers for rat actin mRNA expression (used as a control for total RNA content for each sample) were 5’-TGAAGGTCGGTGTGAACGGATTTGGC-3’ and 5’-CATGTAGGCCATGAGGTCCACCAC-3’ , respectively.

### NF-κB secretory alkaline phosphatase (SEAP) reporter gene assay in transfected-RAW 264.7 cells

The NF-κB SEAP inhibitory activities of calipteryxin and (3*’S*,4*’S*)-3’ ,4’-disenecioyloxy-3’ ,4’-dihydroseselin were determined in LPS-stimulated RAW 264.7 macrophages. The NF-κB-dependent reporter gene transcription was analyzed using the SEAP assay as previously described, with some modifications [5, 7].

### Electrophoretic mobility shift assay (EMSA)

EMSA was performed to investigate the inhibitory effects on NF-κB and AP-1 DNA binding, as previously described [5, 7].

### Computational methods

Docking studies were performed using Glide XP in Schrödinger suite (version 2013).

### Statistical analysis

Unless otherwise stated, the results are expressed as the means ± standard deviations (SD) from three different experiments. One-way analysis of variance (ANOVA) followed by Dunnett’s *t*-test was applied to assess the statistical significance of the differences between the study groups (SPSS version 10.0, Chicago, IL). A value of *p* <0.05 was considered statistically significant.

## Results

Effects of calipteryxin and (3’*S*,4’*S*)-3’ ,4’-disenecioyloxy-3’ ,4’-dihydroseselin on cell viability in LPS- and SNP-induced macrophages

**Fig. 2:**
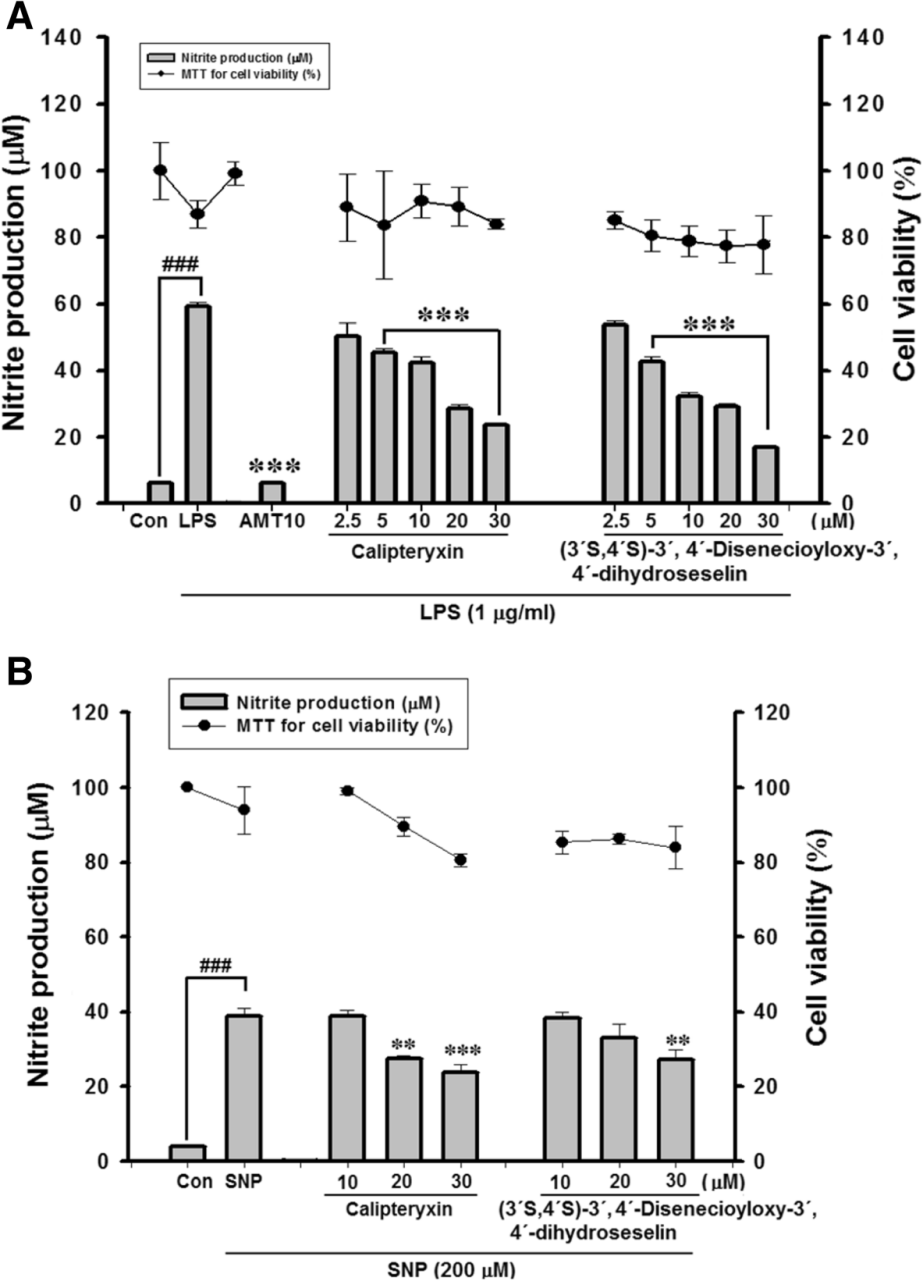
Effects of calipteryxin and (3*’S*,4*’S*)-3’,4’-disenecioyloxy-3’,4’-dihydroseselin on cell viability and NO production in LPS- (**a**) and SNP- (**b**) stimulated-RAW 264.7 cells as described in the “Methods”. The data were derived from three independent experiments and are expressed as the means ± S.D. (***) *p <0.001* indicates a significant difference from the LPS-challenged group. (^*###*^) *p <0.001* indicates a significant difference from the unstimulated control group. Control (vehicle), LPS; (LPS + vehicle)-treated cells alone

The cell viability of calipteryxin and (3’*S*,4’*S*)-3’ ,4’-disenecioyloxy-3’,4’-dihydroseselin was evaluated in LPS- and SNP-stimulated RAW264.7 cells (Fig. 2). No cytotoxic effect was observed in LPS-stimulated macrophages until treatment with a 30-μM concentration of the derivatives. Consequently, non-toxic concentrations were used for the following experiments.

Inhibitory effects of calipteryxin and (3’*S*,4’*S*)-3’,4’-disenecioyloxy-3’,4’-dihydroseselin on NO production in LPS- and SNP-induced RAW 264.7 macrophages

To determine NO production, we measured the amount of nitrite released into the culture medium. RAW 264.7 cells were treated with various concentrations of calipteryxin and (3*’S*,4*’S*)-3’,4’-disenecioyloxy-3’,4’-dihydroseselin (2.5 ~ 30 μM). Incubation with LPS alone markedly increased NO production from these cells, compared with the NO production generated under the control conditions (Fig. 2). However, pre-treatment with calipteryxin and (3*’S*,4*’S*)-3’,4’-disenecioyloxy-3’,4’-dihydroseselin prevented this increased level of NO production in LPS-stimulated RAW 264.7 cells in a concentration-dependent manner (Fig. 2a)., However, SNP-induced NO production was slightly reduced after treatment with calipteryxin and (3*’S*,4*’S*)-3’,4’-disenecioyloxy-3’,4’-dihydroseselin (Fig. 2b). By comparison, calipteryxin and (3’S,4’S)-3’,4’-disenecioyloxy-3’ ,4’-dihydroseselin inhibition was more remarkable in LPS-stimulated macrophages than in SNP-induced macrophages. Therefore, the LPS-stimulated RAW264.7 cell model was used for subsequent in vitro experiments.

Effects of calipteryxin and (3’*S*,4’*S*)-3’,4’-disenecioyloxy-3’,4’-dihydroseselin on LPS-induced iNOS and COX-2 protein and mRNA expression levels

**Fig. 3:**
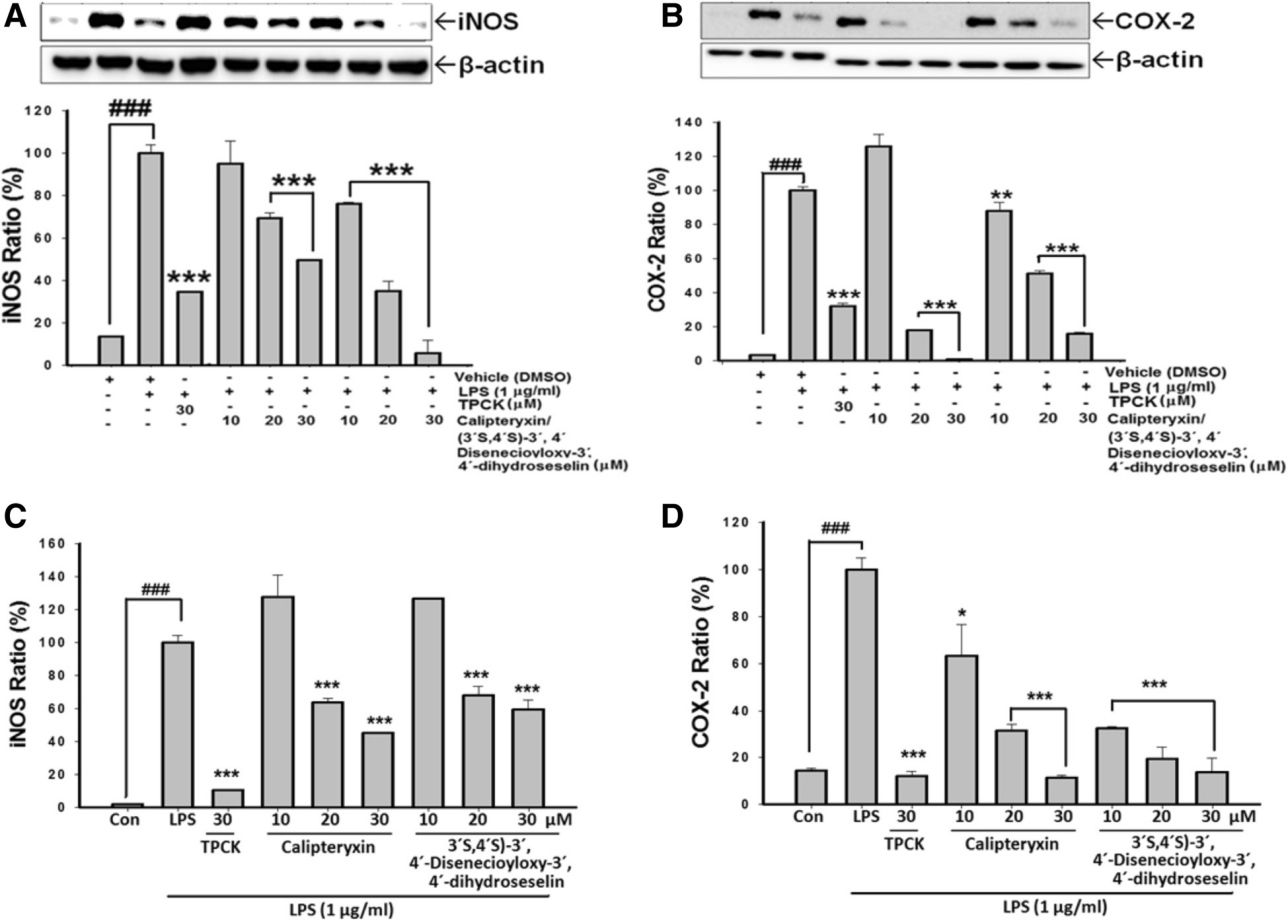
Effects of calipteryxin and (3*’S*,4*’S*)-3’,4’-disenecioyloxy-3’,4’-dihydroseselin on the LPS-induced iNOS protein (**a**), and COX-2 protein (**b**), iNOS mRNA (**c**), COX-2 mRNA (**d**) expression levels in RAW 264.7 macrophages using Western blotting as described in “Methods”. The data were derived from three independent experiments and are expressed as the means ± S.D. (*) *p <0.05*, (**) *p <0.01* and (***) *p <0.001* indicate significant differences from the LPS-challenged group. (^*###*^) *p <0.001* indicates a significant difference from the unstimulated control group. Control (vehicle), LPS; (LPS + vehicle)-treated cells alone; TPCK 30 μM, *N*-*p*-tosyl-L-phenylalanyl chloromethyl ketone was used as a positive control

As calipteryxin and (3*’S*,4*’S*)-3’ ,4’-disenecioyloxy-3’ ,4’-dihydroseselin inhibit NO production, we examined the relationship between the protein and mRNA expression levels of iNOS and COX-2 (Fig. 3). The inhibitory effects of calipteryxin and (3’*S*,4’*S*)-3’ ,4’-disenecioyloxy-3’ ,4’-dihydroseselin on the protein and mRNA expression levels of iNOS and COX-2 were determined through Western blotting and qRT-PCR analyses, respectively. The iNOS and COX-2 protein and mRNA expression levels were markedly up-regulated after LPS treatment, and calipteryxin and (3*’S*,4*’S*)-3’ ,4’-disenecioyloxy-3’ ,4’-dihydroseselin significantly attenuated iNOS and COX-2 mRNA expression in LPS-stimulated macrophages in a concentration-dependent manner (Fig. 3).

**Fig. 4:**
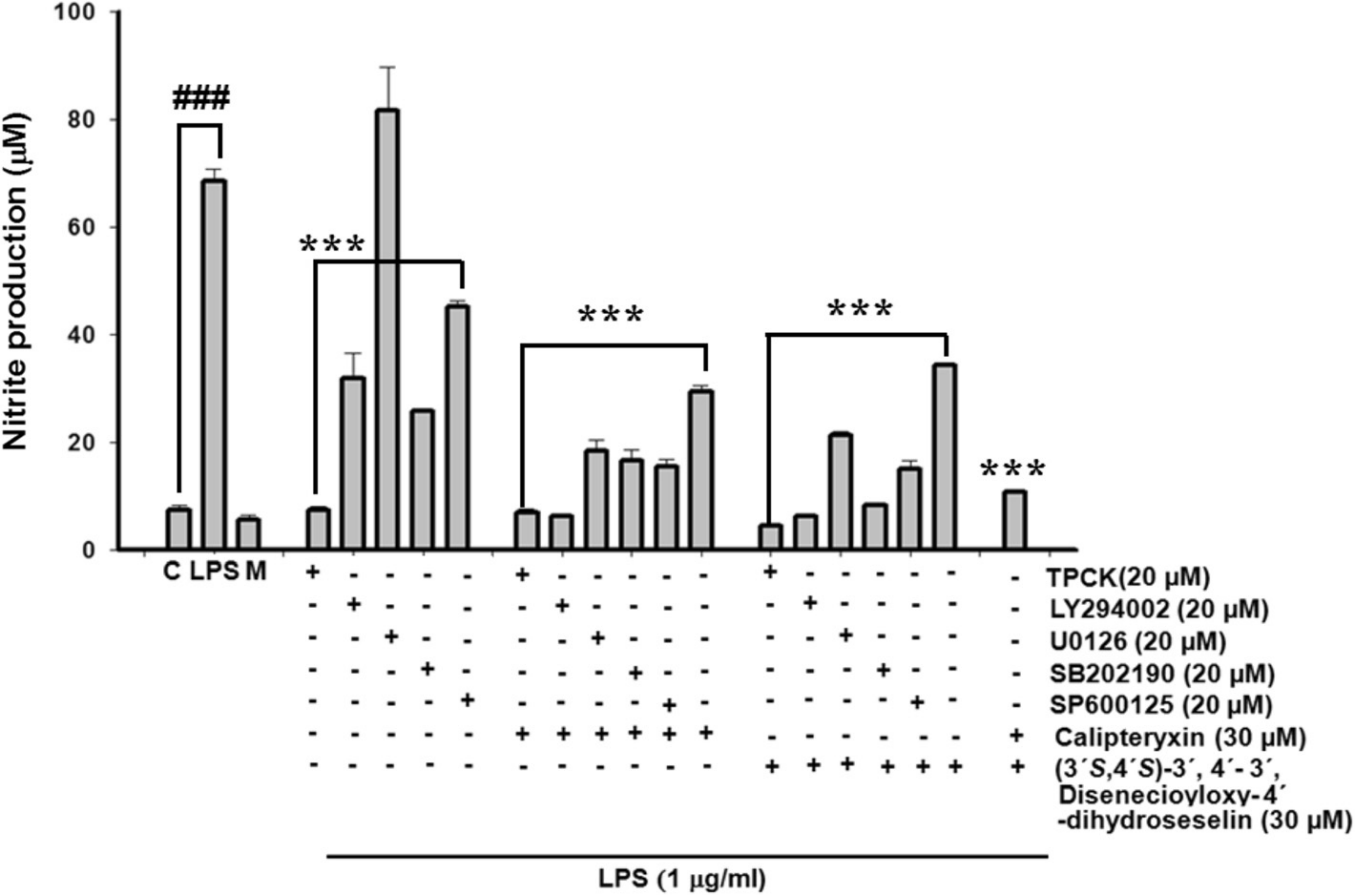
Effects of calipteryxin and (3*’S*,4*’S*)-3’,4’-disenecioyloxy-3’,4’-dihydroseselin on various inhibitors induced by LPS in RAW 264.7 macrophages as described in “Methods.” The data were derived from three independent experiments and are expressed as the means ± S.D. (*) *p <0.05*, (**) *p <0.01* and (***) *p <0.001* indicate significant differences from the LPS-challenged group. (^*###*^) *p <0.001* indicates a significant difference from the unstimulated control group. Control (vehicle), LPS; (LPS + vehicle)-treated cells alone, M; only cells in the media

Effects of calipteryxin and (3’*S*,4’*S*)-3’ ,4’-disenecioyloxy-3’ ,4’-dihydroseselin on LPS-induced NO production using various inhibitors in macrophages

To investigate the inflammatory signaling pathway involved in the inhibitory effects of calipteryxin and (3’*S*,4’*S*)-3’ ,4’-disenecioyloxy-3’ ,4’-dihydroseselin on LPS-induced inflammatory mediators, specific inhibitors of the NF-κB (TPCK, 20 μM), MAPKs (SB202190, p38 MAPK inhibitor; SP600125, JNK inhibitor; U0126, ERK inhibitor) and Akt (LY294002) were used (Fig. 4). The pretreatment of RAW 264.7 cells with TPCK, SB202190, SP600125, and LY294002 significantly inhibited LPS-induced nitrite production in the media, while U0126 showed no effects at 20 μM (Fig. 4). The combination of calipteryxin and (3’*S*,4’*S*)-3’ ,4’-disenecioyloxy-3’ ,4’-dihydroseselin with TPCK and specific inhibitors against p38, JNK, ERK and Akt significantly inhibited LPS-induced NO production (Fig. 4). Overall, these results suggest that p38, JNK, ERK and Akt, in conjunction with NF-κB inflammatory signaling, might contribute to the inhibitory effects of calipteryxin and (3’*S*,4’*S*)-3’ ,4’-disenecioyloxy-3’ ,4’-dihydroseselin on inflammatory mediators.

Inhibitory effects of calipteryxin and (3’*S*,4’*S*)-3’ ,4’-disenecioyloxy-3’ ,4’-dihydroseselin on NF-κB signaling

**Fig. 5:**
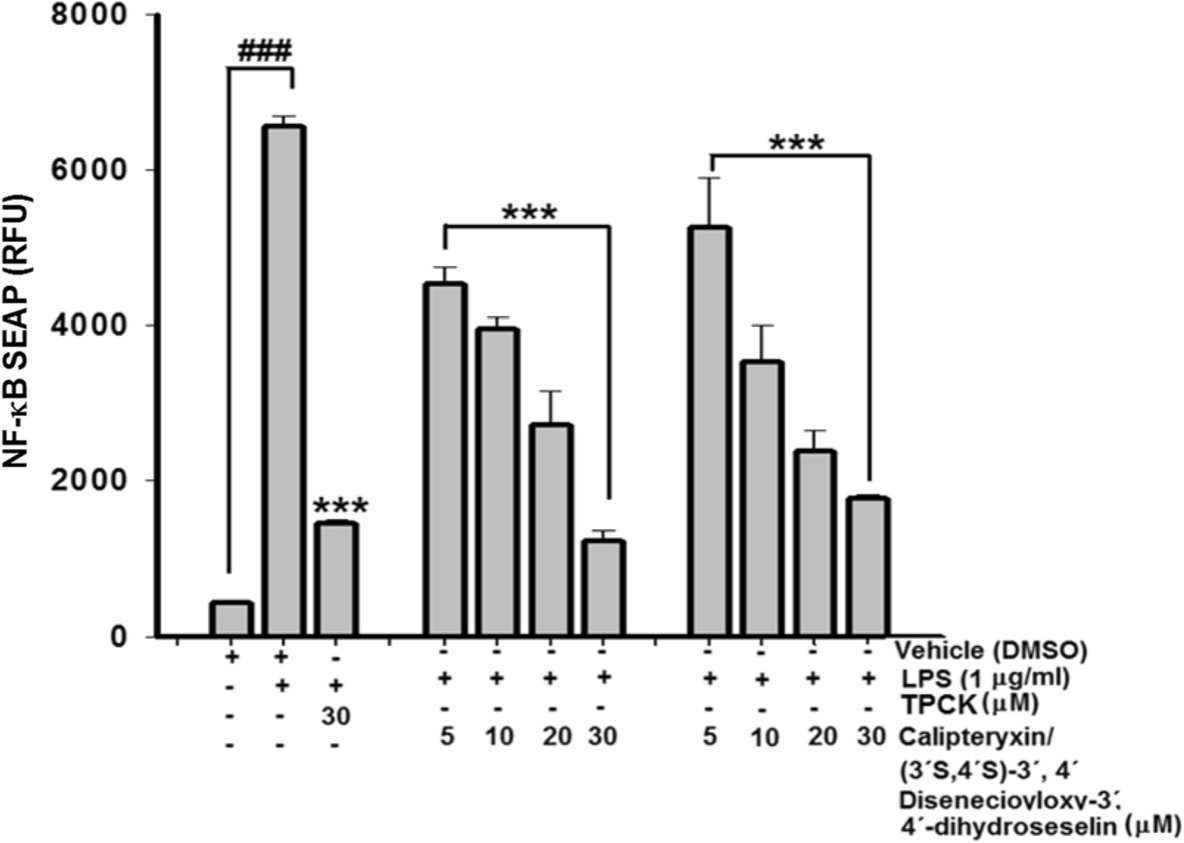
Effects of calipteryxin and (3*’S*,4*’S*)-3’,4’-disenecioyloxy-3’,4’-dihydroseselin on LPS-induced and NF-κB-dependent alkaline phosphatase (SEAP) expression in transfected-RAW 264.7 macrophages as described in “Methods.” The data were derived from three independent experiments and are expressed as the means ± S.D. (***) *p <0.001* indicates a significant difference from the LPS-challenged group. (^*###*^) *p <0.001* indicates a significant difference from the unstimulated control group. Control (vehicle), LPS; (LPS + vehicle)-treated cells alone; TPCK 30 μM, *N*-*p*-tosyl-L-phenylalanyl chloromethyl ketone was used as a positive control

Because the results indicated that calipteryxin and (3*’S*,4*’S*)-3’ ,4’-disenecioyloxy-3’ ,4’-dihydroseselin affect iNOS and COX-2 induction, we focused on two transcription factors critical in iNOS and COX-2 induction, i.e., NF-κB and AP-1 [5, 12]. Initially, the inhibitory effects of calipteryxin and (3*’S*,4*’S*)-3’ ,4’-disenecioyloxy-3’ ,4’-dihydroseselin on NF-κB in the medium were evaluated. As shown in Fig. 5, both compounds exhibited remarkable inhibitory effects on NF-κB in the culture media.

**Fig. 6:**
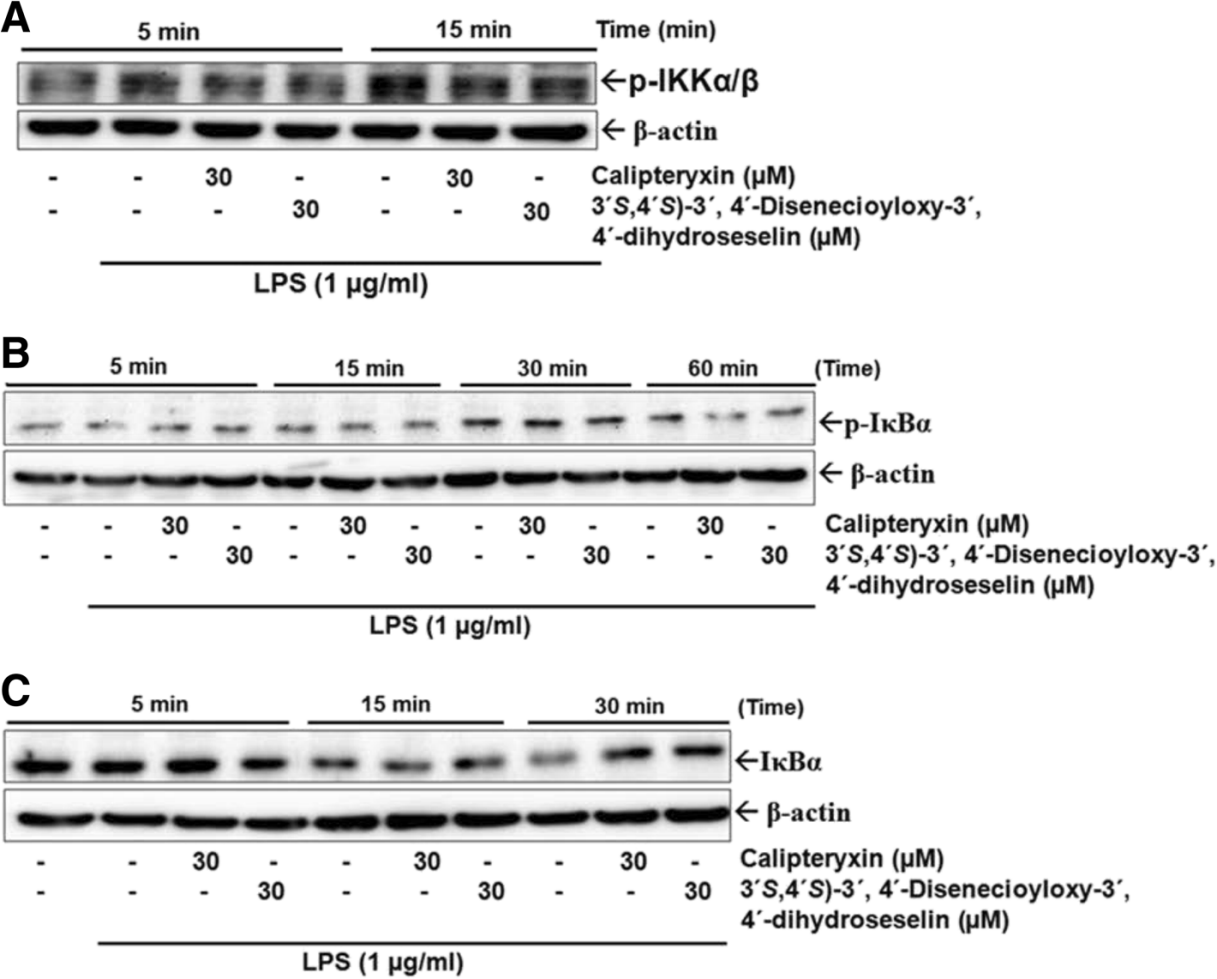
Effects of calipteryxin and (3’S,4’S)-3’,4’-disenecioyloxy-3’,4’-dihydroseselin on p-IKKα/β (**a**), p-IκBα (**b**), and IκBα (**c**) protein expression levels in time course experiments

To analyze whether calipteryxin and (3*’S*,4*’S*)-3’ ,4’-disenecioyloxy-3’ ,4’-dihydroseselin suppressed the phosphorylation and degradation of IκBα and NF-κB nuclear translocation, time course experiments were performed in LPS-stimulated macrophages using Western blot analysis (Fig. 6). After treatment with LPS alone for 1 h, IκBα activation levels were markedly increased, and calipteryxin and (3*’S*,4*’S*)-3’ ,4’-disenecioyloxy-3’ ,4’-dihydroseselin) significantly blocked LPS-induced IκBα phosphorylation after 60 min of LPS stimulation (Fig. 6). However, IκBα degradation through calipteryxin and (3*’S*,4*’S*)-3’ ,4’-disenecioyloxy-3’ ,4’-dihydroseselin was inhibited after 15 min of LPS (Fig. 6). Complete inhibition was observed after 30 min of LPS stimulation (Fig. 6).

In an attempt to explore the effects of calipteryxin and (3*’S*,4*’S*)-3’ ,4’-disenecioyloxy-3’ ,4’-dihydroseselin on the inhibition of IκB kinase (IKK) activity in RAW264.7 cells, Western blot analysis was performed to measure the phosphorylation of IKKα/β after treatment with calipteryxin and (3*’S*,4*’S*)-3’ ,4’-disenecioyloxy-3’ ,4’-dihydroseselin (Fig. 6). Calipteryxin and (3*’S*,4*’S*)-3’ ,4’-disenecioyloxy-3’ ,4’-dihydroseselin significantly inhibited LPS-induced activation of IKKα/β (Fig. 6).

**Fig. 7:**
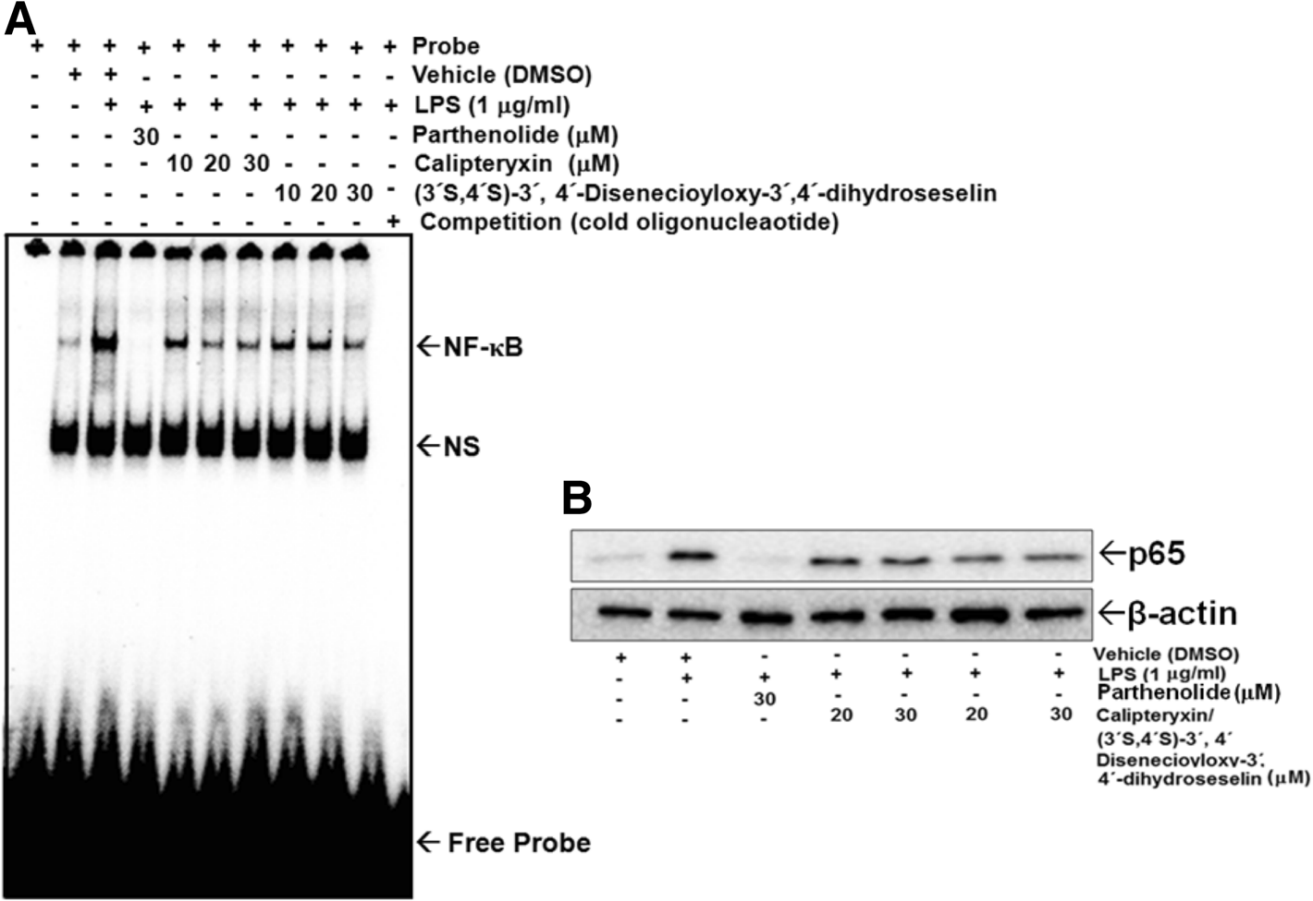
Effects of calipteryxin and (3*’S*,4*’S*)-3’,4’-disenecioyloxy-3’,4’-dihydroseselin on NF-κB-DNA binding activity (**a**) and p65 (**b**) protein expression

**Fig. 8:**
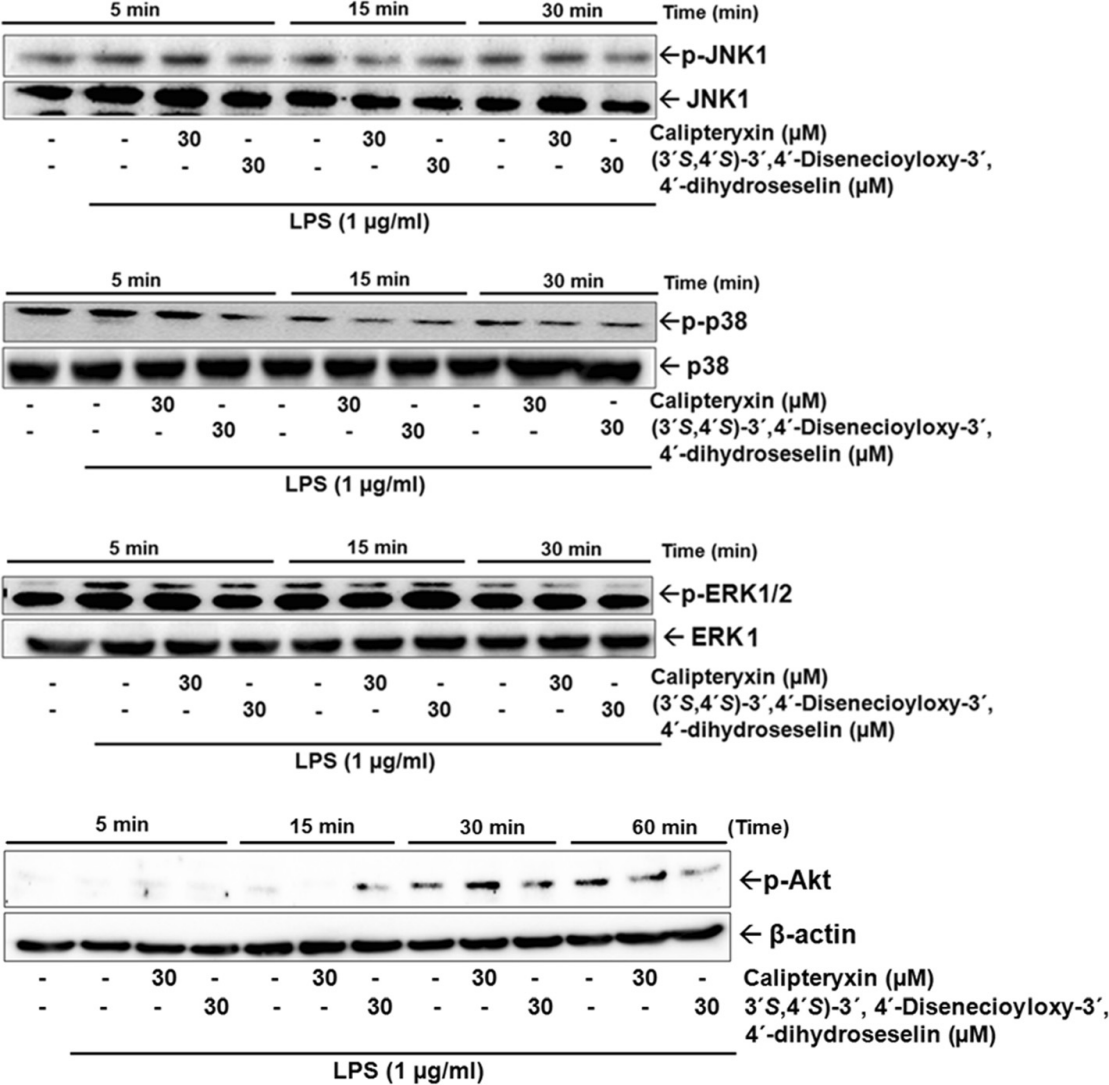
Effects of calipteryxin and (3*’S*,4*’S*)-3’,4’-disenecioyloxy-3’,4’-dihydroseselin on the protein expression levels of the MAPKs p-JNK1, p-p38, p-ERK and p-Akt in time course experiments

Additionally, we examined the DNA-binding affinity of these transcription factors using EMSA (Fig. 7a). Both Calipteryxin and (3*’S*,4*’S*)-3’ ,4’-disenecioyloxy-3’ ,4’-dihydroseselin attenuated the LPS-induced DNA binding activity of both NF-κB (Fig. 7a). The specificity of the bands was confirmed after adding a 50-fold excess of unlabeled NF-κB oligonucleotide to the binding reaction (Fig. 7a). Additionally, the nuclear translocation of p65, a component of the NF-κB heterodimer, was further evaluated (Fig. 8b). LPS induced the translocation of p65 from the cytoplasm to the nucleus after treatment for 1 h, and calipteryxin and (3*’S*,4*’S*)-3’ ,4’-disenecioyloxy-3’ ,4’-dihydroseselin markedly prevented the nuclear translocation of p65 (Fig. 7).

Effects of calipteryxin and (3’*S*,4’*S*)-3’ ,4’-disenecioyloxy-3’ ,4’-dihydroseselin on LPS-induced MAPKs and Akt activations in macrophages

MAP kinases are important for the expression of both iNOS and COX-2 [13, 14]. Thus, MAPKs act as specific targets in inflammatory responses. Because MAPK activation plays an important role in NF-κB stimulation, we examined whether MAPK’s activity is inhibited through treatment with calipteryxin and (3’*S*,4’*S*)-3’ ,4’-disenecioyloxy-3’ ,4’-dihydroseselin. Herein, we assessed the phosphorylation levels of MAP kinases, including JNK1, p38 and ERK 1/2. When RAW 264.7 cells were stimulated with LPS, in the presence of calipteryxin and (3’*S*,4’*S*)-3’ ,4’-disenecioyloxy-3’ ,4’-dihydroseselin, the levels of phosphorylated JNK1, p38 and ERK 1/2 MAPK were observed to significantly start decreasing after 15 min of LPS stimulation (Fig. 8). Additionally, Akt activation was significantly inhibited after treatment with calipteryxin and (3’*S*,4’*S*)-3’ ,4’-disenecioyloxy-3’ ,4’-dihydroseselin after 60 min of LPS stimulation (Fig. 8). These results suggest that the MAPK and Akt pathways are relevant during the LPS-mediated expression of iNOS and COX-2.

Effects of calipteryxin and (3’*S*,4’*S*)-3’ ,4’-disenecioyloxy-3’ ,4’-dihydroseselin on LPS-induced AP-1-DNA binding and c-Jun expression in macrophages

**Fig. 9:**
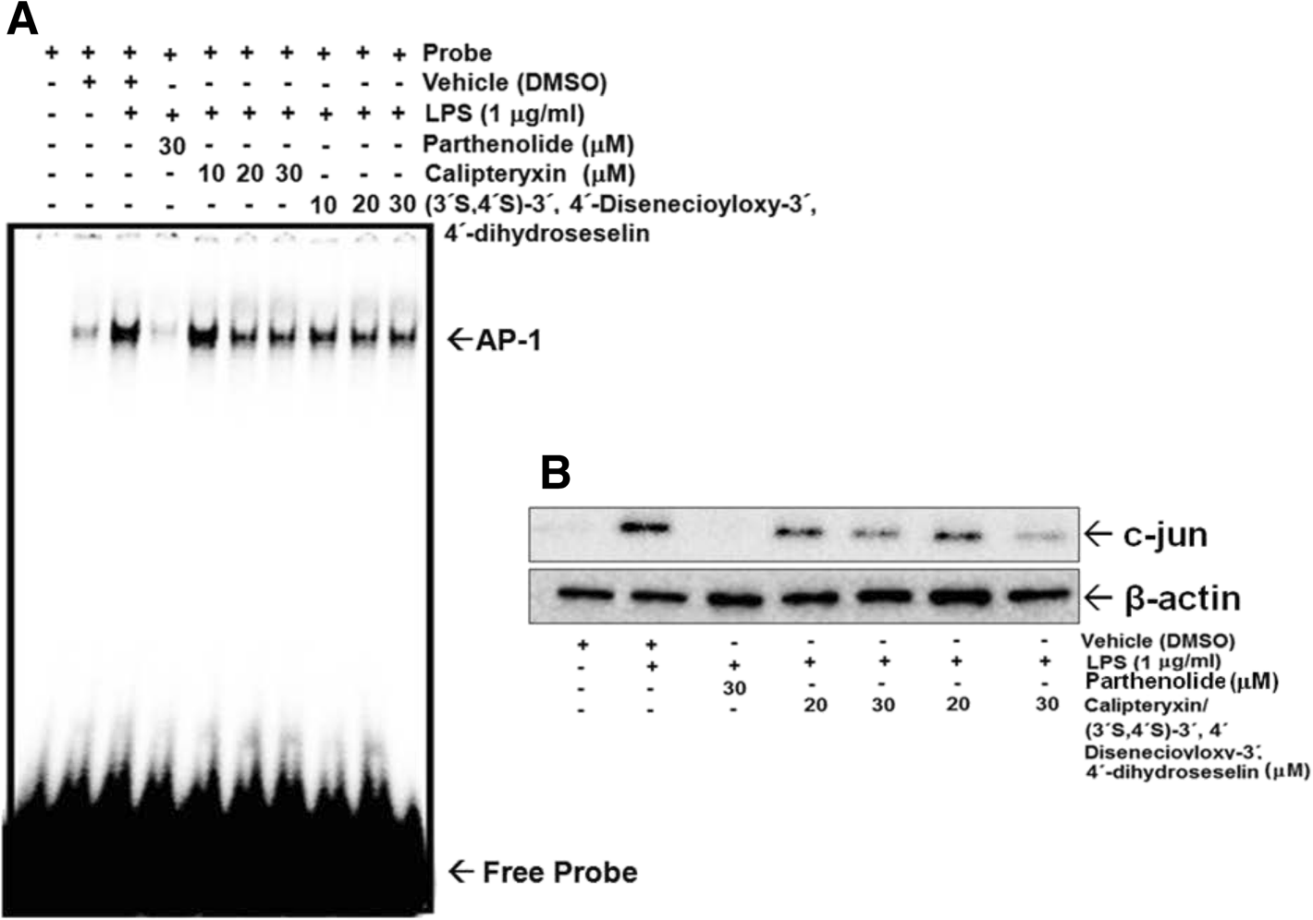
Effects of calipteryxin and (3*’S*,4*’S*)-3’,4’-disenecioyloxy-3’,4’-dihydroseselin on Ap-1-DNA binding activity (**a**) and c-Jun (**b**) protein expression

AP-1 is another transcription factor involved in the regulation of inflammatory processes [13]. To evaluate the effects of calipteryxin and (3’*S*,4’*S*)-3’ ,4’-disenecioyloxy-3’ ,4’-dihydroseselin, we performed EMSA. The results clearly demonstrated that calipteryxin and (3’*S*,4’*S*)-3’ ,4’-disenecioyloxy-3’ ,4’-dihydroseselin remarkably inhibited AP-1-DNA binding activity (Fig. 9), while LPS-stimulated cells showed significantly high DNA-binding affinity (Fig. 9). In addition, the effects of calipteryxin and (3’*S*,4’*S*)-3’ ,4’-disenecioyloxy-3’ ,4’-dihydroseselin on c-jun were considerably promising (Fig. 9).

Effects of calipteryxin and (3’*S*,4’*S*)-3’ ,4’-disenecioyloxy-3’ ,4’-dihydroseselin on pro-inflammatory cytokines in LPS-stimulated macrophages

**Fig. 10:**
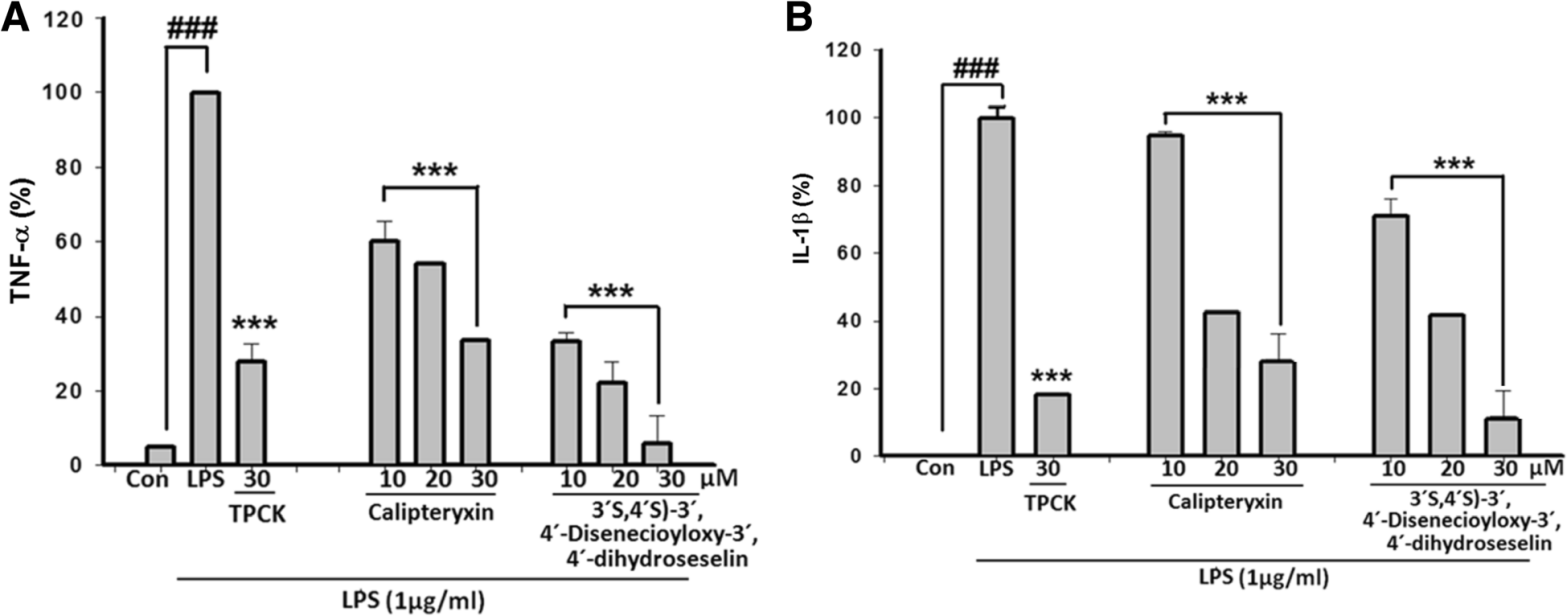
Suppression effects of calipteryxin and (3*’S*,4*’S*)-3’,4’-disenecioyloxy-3’,4’-dihydroseselin on the mRNA expression of the pro-inflammatory cytokines TNF-α (**a**) and IL-1β (**b**). Total RNA was isolated, and the expression of TNF-α and IL-1β was determined through qRT-PCR, as described in the “Methods.” Con (vehicle), LPS; (LPS + vehicle)-treated cells alone and TPCK (30 μM) served as a positive control. (***) *p <0.00*1 indicates significant differences from the LPS-treated group. (^*###*^) *p <0.001* indicates a significant difference from the unstimulated control group

As calipteryxin and (3*’S*,4*’S*)-3’ ,4’-disenecioyloxy-3’ ,4’-dihydroseselin inhibit the activation of the two pro-inflammatory transcription factors NF-κB and AP-1, we examined the effects of calipteryxin and (3*’S*,4*’S*)-3’ ,4’-disenecioyloxy-3’ ,4’-dihydroseselin on the expression of pro-inflammatory cytokines using qRT-PCR analysis. TNF-α and IL-1β are predominantly regulated at the transcriptional level, whereby the transcription factors NF-κB and AP-1 play crucial roles [15]. Indeed, the treatment of LPS-activated cells with calipteryxin and (3*’S*,4*’S*)-3’ ,4’-disenecioyloxy-3’ ,4’-dihydroseselin significantly reduced the secretion of TNF-α and IL-1β in RAW 264.7 cells (Fig. 10).

### Binding model analysis

**Fig. 11:**
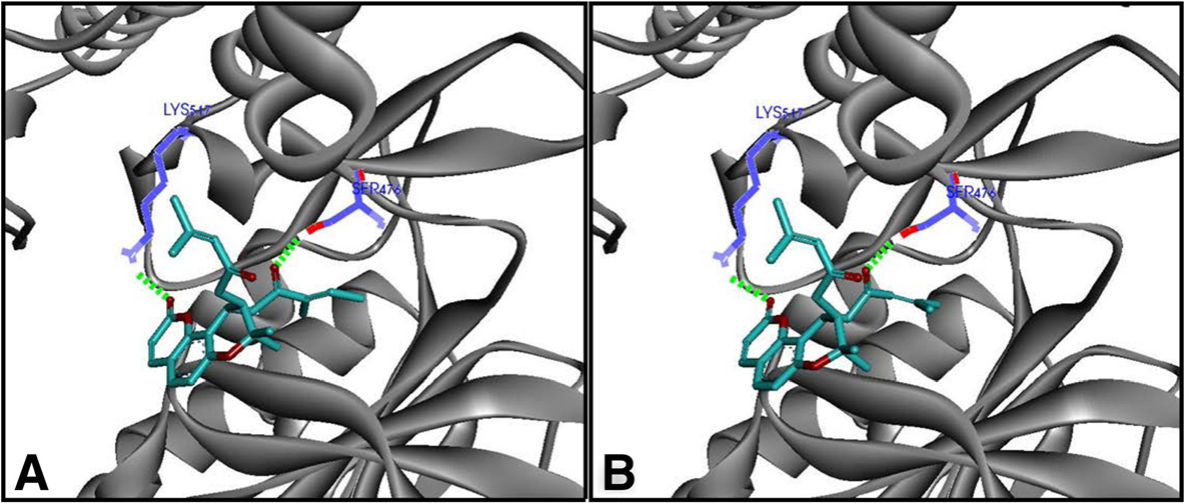
Molecular docking study of calipteryxin (**a**) and (3*’S*,4*’S*)-3’,4’-disenecioyloxy-3’,4’-dihydroseselin (**b**)

To further elucidate the binding of compounds, we performed a docking analysis. Docking was simulated using Glide XP (Schrödinger 2013) to examine the interactions between calipteryxin and (3’S,4’S)-3’ ,4’-disenecioyloxy-3’ ,4’-dihydroseselin in the NIK active site (Fig. 11). Calipteryxin and (3’S,4’S)-3’ ,4’-disenecioyloxy-3’ ,4’-dihydroseselin form two hydrogen bonds with the LYS517 and SER476 residues. The secondary structure of this protein is shown as a solid ribbon (gray). Key residues are displayed in line style (blue), calipteryxin and (3’S,4’S)-3’ ,4’-disenecioyloxy-3’ ,4’-dihydroseselin are displayed in stick style (carbon atoms in cyan), and hydrogen bonds are represented as green dotted lines.

## Discussion

Macrophages serve as a crucial link between innate and adaptive immunity and play pivotal roles in inflammatory signaling [16]. The stimulation of macrophages with bacterial exotoxins, such as LPS, occurs through the specific receptor TLR4 and triggers the recruitment of the cytoplasmic adaptor protein MyD88 and the activation of TIRAP, which subsequently stimulates down-stream signaling pathways (NF-κB and MAPKs). The LPS-induced pathways up-regulate the expression of various inflammatory mediators and cytokines involved in the pathogenesis of inflammatory responses [16]. Based on these hypotheses, the modulation of LPS-induced NF-κB and MAPK signaling or the regulation of cytokine production might constitute a therapeutic strategy in many inflammatory diseases.

Natural products have been one of the leading sources for the discovery of new anti-inflammatory agents. Coumarins are naturally isolated compounds with various remarkable pharmacological and biological properties [8]. In the present study, two different types of coumarins were investigated in terms of LPS-stimulated macrophages through the inhibition of the signaling pathways for the transcription factors NF-κB and AP-1. Initially, the effects of calipteryxin and (3*’S*,4*’S*)-3’ ,4’-disenecioyloxy-3’ ,4’-dihydroseselin on the production of NO and on the regulatory genes for iNOSand COX-2 in LPS-stimulated RAW264.7 macrophages were examined. The LPS-induced down-regulation of the pro-inflammatory mediators through calipteryxin and (3*’S*,4*’S*)-3’ ,4’-disenecioyloxy-3’,4’-dihydroseselin were based on the suppression of the NF-κB and AP-1 signaling, leading to a therapeutic approach against inflammatory diseases.

NO is a free radical produced from *L*-arginine through nitric oxide synthases (NOS) that affects immune functions by eliciting intracellular signals [2, 5]. The high level of NO causes inflammatory damage to target tissue during infection [17]. Hence, the regulation of NO release via the inhibition of iNOS expression is helpful to alleviate inflammatory damage. In the present study, we also showed that calipteryxin and (3*’S*,4*’S*)-3’ ,4’-disenecioyloxy-3’ ,4’-dihydroseselin significantly suppressed LPS-induced-iNOS expression at the transcriptional and translational levels in RAW264.7 cells. Additionally, COX-2 is an inducible isoform of cyclooxygenase that plays an important role in inflammation [18]. Calipteryxin and (3*’S*,4*’S*)-3’ ,4’-disenecioyloxy-3’,4’-dihydroseselin also suppressed LPS-stimulated COX-2 expression in RAW264.7 cells.

We have previously demonstrated that LPS stimulation induces pro-inflammatory enzymes and cytokines through the activation of NF-κB and AP-1 signaling pathways, which play crucial roles in the control of cellular responses to cytokines and stresses [5, 7]. NF-κB is involved in the regulation of the expression of pro-inflammatory cytokines and other mediators involved in the inflammatory response [19]. Therefore, the inhibition of this signaling might explain the potent activity of calipteryxin and (3*’S*,4*’S*)-3’ ,4’-disenecioyloxy-3’ ,4’-dihydroseselin as suppressors of inflammatory cytokines. Under inactive conditions, NF-κB is located in the cytoplasm as an inactive NF-κB/IκBα complex and is controlled through the inhibitory protein IκBα. The degradation of IκBα through phosphorylation releases NF-κB for translocation into the nucleus, thereby initiating the transcription of target genes [2]. Therefore, the translocation of NF-κB could be evaluated in RAW 264.7 cells based on NF-κB-DNA binding affinity. In the present study, LPS induced a marked increase of NF-κB DNA binding, while treatment with the coumarin derivatives significantly inhibited NF-κB-DNA binding activity.

To explore a more in-depth mechanism of calipteryxin and (3*’S*,4*’S*)-3’ ,4’-disenecioyloxy-3’ ,4’-dihydroseselin, MAPKs and Akt signaling pathways were examined. The results of this study demonstrated that calipteryxin and (3*’S*,4*’S*)-3’ ,4’-disenecioyloxy-3’ ,4’-dihydroseselin block the activation of MAPKs and Akt during early LPS stimulation. This significant inhibition might reflect the potential anti-inflammatory effect of calipteryxin and (3*’S*,4*’S*)-3’ ,4’-disenecioyloxy-3’ ,4’-dihydroseselin. Experiments demonstrating the molecular docking of NF-κB inducing kinase (NIK) further supported the molecular analysis data [4]. NIK is a key regulator of inflammation through the non-canonical NF-κB pathway. In response to inflammation, the molecular docking molecule (NIK) in the non-canonical pathway recruits IKKα to p100 for subsequent phosphorylation, ubiquitination and degradation, resulting in the release and translocation of NF-κB heterodimers [4]. The docking simulation revealed that calipteryxin and (3’S,4’S)-3’ ,4’-disenecioyloxy-3’ ,4’-dihydroseselin form two hydrogen bonds with LYS517 and SER476 residues. The secondary structure of the protein is shown as a solid ribbon (gray). Key residues are displayed in line style (blue). Calipteryxin and (3’S,4’S)-3’ ,4’-disenecioyloxy-3’ ,4’-dihydroseselin are displayed in stick style (carbon atoms in cyan).

In addition, the transcription factor AP-1 was inhibited through treatment with both compounds, suggesting that calipteryxin and (3*’S*,4*’S*)-3’ ,4’-disenecioyloxy-3’ ,4’-dihydroseselin suppresses the activation of NF-κB and AP-1/(MAPKs), indicating that the NF-κB pathway is also involved in the anti-inflammatory effects of calipteryxin and (3*’S*,4*’S*)-3’ ,4’-disenecioyloxy-3’ ,4’-dihydroseselin.

## Conclusion

The anti-inflammatory effects of calipteryxin and (3*’S*,4*’S*)-3’ ,4’-disenecioyloxy-3’ ,4’-dihydroseselin were associated with the inhibition of inflammatory enzymes (iNOS and COX-2) and cytokines (TNF-α and IL-1β) via NF-κB, MAPK and Akt pathways. These results suggest that calipteryxin and (3*’S*,4*’S*)-3’ ,4’-disenecioyloxy-3’ ,4’-dihydroseselin represent a potential alternative treatment for inflammation as well, and provide additional clarification of the underlying molecular mechanisms. More in-depth studies are required for the detailed investigation of the molecular mechanisms and structure activity relationships involving these molecules.

## Additional file

Additional file 1: Figure S1. Time optimization of coumarins treatment at various time point by stimulating LPS in RAW 264.7 cells.
