# Supplementary material for: Pharmacological mechanism underlying anti-inflammatory properties of two structurally divergent coumarins through the inhibition of pro-inflammatory enzymes and cytokines
Source: J Inflamm (Lond). 2015 Jul 29;12:47. doi: 10.1186/s12950-015-0087-y (PMC4517569; doi:10.1186/s12950-015-0087-y)
Supplement: Additional file 1: Figure S1. — Time optimization of coumarins treatment at various time point by stimulating LPS in RAW 264.7 cells. [file 12950_2015_87_MOESM1_ESM.docx]

**Supplimentary**

Pharmacological mechanism underlying anti-inflammatory properties of two structurally divergent coumarins through the inhibition of pro-inflammatory enzymes and cytokines

Salman Khan^1,2^, Omer Shehzad^2,3^, Mao-Sheng Cheng^4^, Rui-Juan Li^4^, and Yeong Shik Kim^2^*

*^1^The Alan Edwards Centre for Research on Pain, McGill University, Montreal, Quebec H3A 0G1 Canada*

*^2^College of Pharmacy, Seoul National University, Seoul 151-742, South Korea*

*^3^Department of Pharmacy, Abdul Wali Khan University, Mardan, Pakistan*

*^4^School of Pharmaceutical Engineering, Shenyang Pharmaceutical University, Shenyang 110016, China*

***Corresponding Author**: Prof. Yeong Shik Kim, College of Pharmacy, Seoul National University, Gwanak-gu, Seoul 151-742, South Korea.

Tel: +82-2-8802479. Fax: +82-2-7654768.

E-mail: [kims@snu.ac.kr](mailto:kims@snu.ac.kr)


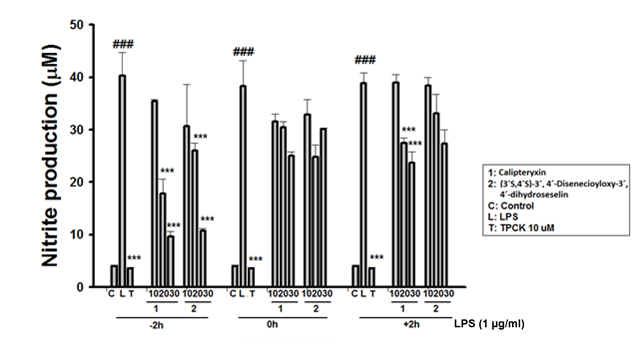


**Fig S1: Time optimization of coumarins treatment at various time point by stimulating LPS in RAW 264.7 cells.**
